# Exploring the depths of IgG4: insights into autoimmunity and novel treatments

**DOI:** 10.3389/fimmu.2024.1346671

**Published:** 2024-04-18

**Authors:** Selen Ünlü, Blanca G. Sánchez Navarro, Elif Cakan, Daniel Berchtold, Rafael Meleka Hanna, Secil Vural, Atay Vural, Andreas Meisel, Miriam L. Fichtner

**Affiliations:** ^1^ Koç University Research Center for Translational Medicine (KUTTAM), İstanbul, Türkiye; ^2^ Koç University School of Medicine, Istanbul, Türkiye; ^3^ Department of Neurology with Experimental Neurology, Integrated Myasthenia Gravis Center, Neuroscience Clinical Research Center, Charité Universitätsmedizin Berlin, Berlin, Germany; ^4^ Ragon Institute of Massachusetts General Hospital, Massachusetts Institute of Technology and Harvard University, Cambridge, MA, United States; ^5^ Department of Dermatology and Venereology, Koç University School of Medicine, İstanbul, Türkiye; ^6^ Department of Neurology, Koç University School of Medicine, İstanbul, Türkiye

**Keywords:** IgG4, IgG4-AID, IgG4-RD, immunotherapies, antibodies

## Abstract

IgG4 subclass antibodies represent the rarest subclass of IgG antibodies, comprising only 3-5% of antibodies circulating in the bloodstream. These antibodies possess unique structural features, notably their ability to undergo a process known as fragment-antigen binding (Fab)-arm exchange, wherein they exchange half-molecules with other IgG4 antibodies. Functionally, IgG4 antibodies primarily block and exert immunomodulatory effects, particularly in the context of IgE isotype-mediated hypersensitivity reactions. In the context of disease, IgG4 antibodies are prominently observed in various autoimmune diseases combined under the term IgG4 autoimmune diseases (IgG4-AID). These diseases include myasthenia gravis (MG) with autoantibodies against muscle-specific tyrosine kinase (MuSK), nodo-paranodopathies with autoantibodies against paranodal and nodal proteins, pemphigus vulgaris and foliaceus with antibodies against desmoglein and encephalitis with antibodies against LGI1/CASPR2. Additionally, IgG4 antibodies are a prominent feature in the rare entity of IgG4 related disease (IgG4-RD). Intriguingly, both IgG4-AID and IgG4-RD demonstrate a remarkable responsiveness to anti-CD20-mediated B cell depletion therapy (BCDT), suggesting shared underlying immunopathologies. This review aims to provide a comprehensive exploration of B cells, antibody subclasses, and their general properties before examining the distinctive characteristics of IgG4 subclass antibodies in the context of health, IgG4-AID and IgG4-RD. Furthermore, we will examine potential therapeutic strategies for these conditions, with a special focus on leveraging insights gained from anti-CD20-mediated BCDT. Through this analysis, we aim to enhance our understanding of the pathogenesis of IgG4-mediated diseases and identify promising possibilities for targeted therapeutic intervention.

## 1 Introduction

A prerequisite for selecting effective therapies is a profound understanding of the underlying (immuno-) pathology. Many existing therapies, while exhibiting efficacy, are often expensive and may induce side effects that significantly compromise the overall quality of life for patients (1). Therefore, a deeper understanding of the immunopathology is necessary to make informed treatment decisions and to identify therapies that are both effective and efficient in a personal-tailored manner. To gain deeper insights into immunopathology, a valuable approach is to employ reverse translational medicine. In reverse translational medicine, scientific discoveries are informed by clinical observations (2). The clinical observation this review is based on is the remarkable effect of anti-CD20-mediated B cell depletion therapy (BCDT) in disorders with a prevalence of IgG4 subclass antibodies (3–12). This effect is not exclusive for IgG4-mediated diseases where antibodies are the major effectors of pathology like in IgG4 autoimmune diseases (IgG4-AID); IgG4-related disease (IgG4-RD) also responds well to anti-CD20-mediated BCDT (13). In this review will first explore B cells, antibody subclasses, and their properties in general, before we specifically highlight the unique features of IgG4 subclass antibodies in the context of IgG4-AID and IgG4-RD. In the concluding segment of this review, we will examine potential therapies for these diseases, with a particular focus on exploring insights derived from anti-CD20-mediated BCDT.

## 2 Insights into B cell functions and antibody diversity

In autoimmune diseases, the immune system malfunctions, targeting the body’s own structures. While the immune response involves a variety of cells, some of these autoimmune diseases are characterized by a prominent role of B cells and their effector molecules - the autoantibodies. B cells originate in the bone marrow and undergo several stages of development before maturing into antibody-secreting cells (ASCs), namely plasmablasts and plasma cells (14, 15). The distinct developmental stages of B cell subsets can be identified by surface markers that are expressed at varying levels throughout the maturation process (16). These surface markers are the basis for several B cell targeting therapies, which we will further explore in the section on therapeutic interventions.

In addition to their function as ASCs, B cells play diverse roles in the immune system. They contribute to antigen presentation, cytokine secretion, and the regulation of immune responses (17–19). B cells play a crucial role in modulating T cell responses in both health and disease. Both B and T cells originate from common precursors in the bone marrow, but T cells undergo their final maturation in the thymus (20). B and T cells constitute the adaptive immune system. While B cells contribute to the humoral immune response, T cells serve as the effectors of the cellular response (15). Through antigen presentation, B cells contribute to the negative selection of autoreactive T cells in the thymus, regulate the extent of primary CD4+ T cell responses, and contribute to T cell memory generation (21–23). Antigen presentation by B cells contributes to the immunopathology in several T cell-mediated diseases, including autoimmune hepatitis, rheumatoid arthritis and multiple sclerosis (24–28). Furthermore, B cells are essential for the formation and maintenance of humoral immunological memory, which is crucial for a rapid and effective response upon re-exposure to pathogens (29–31). These memory B cells constitute a crucial reservoir for the generation of ASCs and the corresponding antibody repertoire (14, 31). Throughout the progression of an immune response to an external antigen, B cells undergo affinity maturation, enhancing the affinity of antibodies they produce (32–34). This heightened affinity arises from the interplay of clonal selection and the somatic hypermutation (SHM) process, leading to the gradual accumulation of antibodies with successively greater affinities.

**Figure 1:**
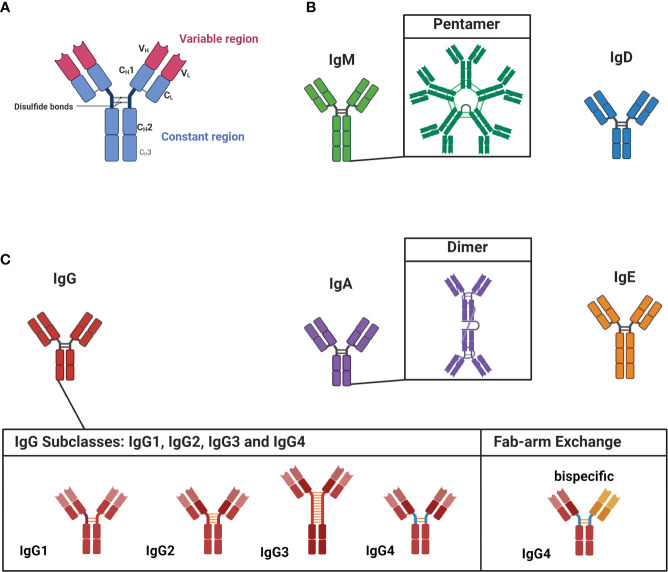
Structure of antibodies, isotypes and IgG subclasses. **(A)** Antibodies are dimeric structures that consist of two identical antigen-binding sites, known as the variable region (VH and VL). This region, called the fragment-antigen binding (Fab) region, is formed by the combination of the light chain and the heavy chain and is the antigen binding site of the antibody. The hinge region connects the Fab region to the constant region (Fc region, CH1-CH3), which determines the antibody’s isotype and function. **(B)** The isotypes of IgM and IgD are associated with less experienced, or “naïve,” cells. IgM is found as a pentamer. **(C)** IgG, IgA, and IgE are linked to more matured antibodies. IgA has the ability to form dimeric structures, while IgG has four subclasses: IgG1, IgG2, IgG3, and IgG4. IgG4 subclass antibodies can undergo Fab-arm exchange, allowing them to become bispecific. Figure created with Biorender.com.

Within the human serum, there is a constant presence of approximately ≈ 9-15 g/L of various IgG subclass antibodies circulating throughout the body (35). These antibodies are polyclonal which means they display diverse specificities, recognizing individual distinct antigens. The interaction between an antibody and its corresponding antigen is highly specific. The segment of the antibody directly involved in this antibody-antigen interaction is termed the variable region (**Figure 1A**), whereas the constant region - the basis for the categorization of antibodies into isotypes and subclasses - is linked to the antibody’s effector and pathogenic function as well as maturation state (**Figures 1B, C**) (36). Antibodies are categorized into IgD, IgM, IgE, IgA, and IgG isotypes (37). IgD and IgM are primarily linked to B cells during the naive stage, while antigen-experienced B cells predominantly employ the other isotypes (38).

## 3 The divergent properties of IgG1-4 subclass autoantibodies and unique features of IgG4

The diseases highlighted in this review predominantly involve autoantibodies belonging to the IgG subclass, which can be further divided into IgG1, IgG2, IgG3 and IgG4 (37). Each subclass has distinct structural and functional properties, leading to varied effector functions including complement activation, opsonization (= presenting antigens to phagocytes), antibody-dependent cell-mediated cytotoxicity (ADCC) and neutralization of toxins (37). The most abundant subclass is IgG1 (≈ 65-70%) (39); opsonization, ADCC and complement activation are among the predominant effector functions of IgG1 antibodies (37, 40). IgG2 (≈ 20-25%) antibodies, in contrast, are less effective in complement activation, but potent in opsonization of encapsulated bacteria (39–41). The third most common subclass is IgG3 (≈ 5-8%); IgG3 antibodies are very potent effectors of the immune system and activate the complement cascade, induce ADCC and neutralize toxins (39, 41, 42).

Notably, the least common subclass IgG4 (≈ 3-5%) stands out among these subclasses due to its unique ability to undergo the process of fragment antigen binding (Fab)-arm exchange (39, 43). IgG subclass antibodies are normally dimers with two identical binding sites (**Figure 1A**). Fab-arm exchange enables IgG4 subclass antibodies to exchange (half)-molecules with other IgG4 antibodies, resulting in bispecific antibodies with two distinct variable regions (**Figure 1C**; on the left). Fab-arm exchange requires the CH3-domain of the IgG4 antibody (see **Figure 1A**) and a reducing environment. As this reaction does not require any additional proteins or co-factors, it results in a highly dynamic process during which IgG4 antibodies constantly exchange arms *in vivo* which generates an ever-changing repertoire of hetero-bivalent IgG4 antibodies (43). Fab-arm exchanged-bispecific IgG4 antibodies cannot cross-link antigens and do not form immune complexes (37). In addition, structural differences in the CH2 domain of IgG4 antibodies are responsible for its low affinity toward C1q from the complement cascade and activating Fcγ receptors (44, 45). However, a recent study revealed that elevated levels of IgG4 antibodies can activate the complement cascade (46). Both variable and constant region glycosylation were found to influence this process. Nonetheless, the biological relevance of this finding remains uncertain. Thus, IgG4 subclass antibodies are generally considered immunomodulatory and referred to as ‘blocking antibodies’ and might function as ‘antigen sink’ (45, 47–49). The blocking function is evident in the context of insect venom or house dust mite hypersensitivity. Antigen-specific IgG4 block the binding of sensitizing IgE to the allergens and consequently prevent allergic symptoms (50). In a similar manner, IgG4 can also prevent hypersensitivity reactions during chronic helminth infections (51, 52). The role of IgG4 subclass antibodies in IgG4-AID and IgG4-RD is further delineated for each specific condition in the subsequent chapter.

The generation of IgG4 most frequently occurs in situations of chronic antigen exposure and might be associated with a continuous germinal center reaction and consecutive rounds of class switch recombination that eventually terminates with a class switch toward IgG4 (53–55). This distinctive property renders IgG4 antibodies particularly prevalent in conditions involving recurrent antigen exposure, as evident in beekeepers and antibodies recognizing bee-related toxins (47, 56–59). The process of class switch to IgG4, similar to the generation of IgE, is regulated by the cytokines IL-4 and IL-13 and necessitates co-stimulation by CD40 (60). Importantly, IL-10 has been shown to reduce IgE secretion and increase IgG4 production and might be directly involved in regulating class switch recombination toward IgG4 (60). More recently, a specialized subset of follicular T helper (Tfh) cells was discovered in tertiary lymphoid tissues of patients suffering from IgG4-related disease. These Tfh cells express the markers BCL6, CXCR5, and ICOS as well as the cytokines IL-4, IL-21, and IL-10 and therefore might crucially regulate the switch to IgG4 (61).

## 4 Features of autoantibodies in IgG4-mediated diseases

IgG4 subclass antibodies are notably predominant in IgG4-AID and IgG4-RD (3–10). A summary of the clinical and epidemiological characteristics of the diseases can be found in **Table 1**. This review focuses on the immunopathology of these diseases and the properties of the corresponding antibodies in the context of disease.

**Table 1:** Comparison of characteristics of IgG4 subtypes of MG, CIDP, Pemphigus and IgG4-RD.

	MG	Nodo-paranodopathies	Pemphigus	Encephilits	IgG4-RD
**Antigenic Target of IgG4 Subclass Autoantibodies**	MuSK	Neurofascin-155, contactin-1/caspr-1, pan-neurofascins	PV: Desmoglein 1, Desmoglein 3 PF: Desmoglein 1	LGI1, CASPR2	–
**Relevance of Fab-arm exchange**	Increases pathogenicity of autoantibodies	Decreases pathogenicity of autoantibodies	PF: Increase of pathogenic effect PV: monovalent autoantibodies are pathogenic, pathogenic capacity most likely not influenced by valency	–	most likely present; disease not autoantibody-mediated
**Prevalence (per 100,000)**	2.2 to 36.7 (62, 63)	0.8 to 8.9 (64)	0.4 to 30 (65)	LIG1: 8.3 (66) CASPR2: -	0.8 to 1.4 (67)
**Age of Onset**	before the age of 40 (68, 69)	40-60 (70, 71)	PV: 40-60 (72, 73) PF: 50-60 (74)	40-60 (mean age of 43-44) (75, 76)	50-60 (mean age of onset 56,5) (67)
**Male to Female Ratio**	Predominantly female (68, 69)	Predominantly male (64, 77–79)	Predominantly female (PV) (80, 81) Equally distributed (PF) (74)	Predominantly male (75, 82)	58% female (67)
**Genetic Factors**	HLA class II genes: HLA DQB1*05, DRB1*14 and DRB1*16 (83–89)	No clear genetic predisposition 90)	HLA class II genes (72, 91–95)	LIG1: HLA-DRB1*07:01, DQA1*02:01, DQB1*02:02 (96, 97) CASPR2: HLA-DRB1*11:01, DQA1*05:01, DQB1*03:01 (97, 98)	HLA-DRB1 and FCGR2B regions (99)
**Location of Pathology**	Neuromuscular junction	Axon of nerves at the Nodes of Ranvier	Skin and mucous membranes	Brain	No specific site; Autoimmune-mediated fibroinflammatory lesions
**Clinical Presentation**	Muscle weakness, increased fatigability	Progressive Weakness, Numbness	Skin Blisters, Lesions, Rash	LGI1: limbic encephalitis with faciobrachial dystonic seizures CASPR2: limbic encephalitis, Morvan’s syndrome, peripheral nerve hyperexcitability syndrome, ataxia and distinct movement disorders	Organ-specific and Systemic Involvement
**Therapy**	Corticosteroids, PE, iVIG, Immuno-suppressants, RTX	Corticosteroids, PE, iVIG, Immunosuppressants, RTX	Corticosteroids, Immunosuppressants, RTX	Corticosteroids, PE, iVIG, Immuno-suppressants, RTX	Corticosteroids, RTX

MuSK, muscle-specific tyrosine kinase; PF, pemphigus foliaceus; PV, pemphigus vulgaris; PE, plasma exchange; iVIG, intravenous Immunoglobulin; RTX, Rituximab.

### 4.1 MuSK myasthenia gravis

The autoantibodies in myasthenia gravis (MG) target structures at the muscle endplate of the neuromuscular junction (NMJ) hindering neuromuscular transmission (100, 101). These autoantibodies disrupt the interaction between the ligand acetylcholine and the acetylcholine receptor (AChR) at the NMJ. They achieve this by directly targeting the AChR or structures within the muscle-specific tyrosine kinase (MuSK)/Low Density Lipoprotein Receptor-Related Protein 4 (LRP4) pathway, critical for the proper clustering and functionality of AChRs (102). The consequence of this interference is the disruption of signaling from nerves to muscles and patients present clinically with increased fatigability and muscle weakness (100, 101). MG is a very heterogeneous disease consisting of several different subtypes. These subtypes are partly categorized by the target antigen that the antibodies detect. So far, three main target antigens have been identified and validated: AChR (103, 104), MuSK (105), and LRP4 (106, 107). Although all of these subtypes are unified under the term MG, each disease has distinct clinical and immunological features (59). The immunological differences are further apparent in the subclass usage of each subtype. While AChR, and LRP4 autoantibodies are mostly of the IgG1, IgG2 and IgG3 subclass (59, 106, 108), MuSK is predominantly IgG4 (4–6). Interestingly, antibodies of the IgG1-3 subclasses in MuSK MG impact the clustering of AChRs (109, 110), resulting in a reduction of clustered AChRs. The precise mechanism underlying this effect remains to be fully elucidated and seems to be divergent from IgG4 MuSK autoantibodies (110).

The isolation and characterization of monoclonal autoantibodies (mAbs) against MuSK has significantly advanced our understanding of MuSK-MG (12, 111–114). The knowledge that plasmablasts are a source for MuSK autoantibodies and the development of mechanisms for the enrichment of this specific B cell population (111–114), were instrumental for the generation of MuSK mAbs (111, 113, 114). The ectodomain of MuSK consists of three Ig-like (Ig1-3) domains and a frizzled domain (115, 116). Previous findings revealed that the majority of MuSK autoantibodies recognize the Ig-like domain 1 on the MuSK receptor, as observed with human polyclonal sera (117). These polyclonal autoantibodies were proven to be pathogenic *in vitro* and *in vivo* via passive transfer (118–120), directly inhibiting the interaction between MuSK and LRP4 (109, 117). More recently, mAbs detecting the Ig-like domain 2 were also shown to have pathogenic capacities (113). The impact of valency on the pathogenicity after Fab-arm exchange of MuSK autoantibodies was initially demonstrated in sera-based experiments (121) and subsequently confirmed with human MuSK mAbs (112, 122, 123). It has further been shown that affinity maturation plays a crucial role in the pathogenic development of MuSK mAbs and that a high affinity coupled with monovalency is essential to reach a pathogenic threshold necessary for the potent disruption of AChR clusters at the NMJ (122). MuSK autoantibody titer correlates well with disease severity in MuSK MG (5, 6, 124–126), might be a potential biomarker to detect relapse in MuSK MG (114, 127) and treatment success with BCDT (114, 124).

### 4.2 Nodo-paranodopathies with autoantibodies targeting NF155, CNTN1, and CASPR1

Chronic inflammatory demyelinating polyradiculoneuropathy (CIDP) is a progressive autoimmune peripheral neuropathy, where the main target is the myelin sheath of peripheral nerves (128, 129). Over the last decade, antibodies targeting the proteins located in the nodes of Ranvier and the paranodal region have been identified and reported to be present in 2-15% of patients clinically diagnosed with CIDP (10, 130). Although these patients are generally diagnosed clinically as CIDP, there is a growing consensus on classifying seropositive CIDP patients under nodo-paranodopathies instead of CIDP, as there are distinct immunopathological and clinical characteristics between IgG4-mediated nodo-paranodopathies and CIDP (131–133).

These antibodies target mainly neurofascin 155 (NF155), neurofascin 186 (NF186), neurofascin 140 (NF140), contactin-1 (CNTN1), and contactin-associated protein 1 (CASPR1) (8, 10, 134–138). In the majority of patients with nodo-paranodopathy, the dominant immunoglobulin subclass is IgG4, particularly for anti-NF155, anti-CNTN1, and anti-CASPR1 (8, 10). These proteins function as cell adhesion molecules. NF155 proteins are located mainly on the myelin loops of Schwann cells and bind to the CNTN1-CASPR1 complexes that are found on the axolemma. The resulting tripartite protein complexes attach the myelin loops strongly to the axon in the paranodes, resulting in formation of the largest junctions known in the body. Antibodies against CNTN1 and CASPR1 bind to the epitopes found in domains that interact with the partner proteins, therefore abolishing the formation of tripartite complexes and leading to disruption of paranodal junctions, resulting in conduction deficits (10, 139). A distinguished feature of anti-NF155 IgG4 antibodies is that these antibodies do not prevent the interaction of its target protein with its partners. Instead, binding of these antibodies leads to the formation of NF155 clusters on Schwann cell surface, resulting in depletion of proteins necessary for the formation of the paranodal complex (140). In summary, unlike classical CIDP, IgG4 antibodies against paranodal antigens do not cause inflammation or demyelination, but rather cause paranodal detachment and disturbance of nodal electrophysiology leading to conduction blocks and potentially axonal degeneration.

Further examination of patient sera with anti-NF155 antibodies revealed that pathogenic monospecific bivalent IgG4 antibodies are present in the sera. The effect of monovalency on pathogenicity is variable in nodo-paranodopathies. In contrast to MuSK MG, the decreased valency of anti–Neurofascin-155 IgG4 subclass autoantibodies resulting from Fab-arm exchange strongly diminished the effect of pathogenic antibodies (141). In CNTN1 autoantibodies, however, monovalency showed similar pathogenic capacities in comparison to their divalent counterparts (142). Similar to MuSK MG, the autoantibodies titers in nodo-paranodopathies correlate well with clinical diseases severity (137, 143, 144).

### 4.3 Pemphigus with autoantibodies targeting Dsg1 and Dsg2

Pemphigus is a group of autoimmune bullous diseases characterized by a pathogenic autoimmune response, primarily driven by autoantibodies targeting two key desmosomal adhesion proteins (145). Desmogleins are Ca^2+^-dependent transmembrane proteins situated in keratinocyte desmosomes and play a crucial role in maintaining the integrity and cohesion of keratinocytes within the epidermis (146). Dsg1 is primarily located in superficial layers, while Dsg3 is found in basal and parabasal layers of the skin (147). The two main subgroups of pemphigus are pemphigus vulgaris (PV) and pemphigus foliaceus (PF), the latter being less common (3, 7, 9).

Pathogenic autoantibodies in pemphigus are of the IgG1 and IgG4 sublcass. These antibodies specifically identify epitopes situated within the EC1 and EC2 domains of Dsg1 and Dsg3 (148). The interaction of these autoantibodies results in the obstruction of cell-cell adhesion, leading to the development of skin blisters (3, 149). This pathological mechanism has been substantiated *in vitro*, wherein the antibodies prompt the separation of cell sheets in cultured human keratinocytes and human skin explants (150–152). Clinical evidence underscores the pathogenicity of IgG4 in comparison to other serum IgG fractions, as eliminating IgG4 from PV sera has been observed to lead to a 81% reduction in dissociation in keratinocyte assays (7). In cases of active disease, both PV and PF patients typically manifest enriched desmoglein reactive IgG4 and IgG1, while individuals in remission and certain healthy relatives of pemphigus patients may solely exhibit IgG1 (149, 153–155). Additionally, the passive transfer of maternal antibodies to the fetus induces a transient neonatal form of pemphigus [201]. Polyclonality and epitope specificity affect the pathogenic effect of autoantibodies in PV (156). Beyond impeding cell-cell adhesion, the autoantibodies can also modulate signal transduction pathways that influence cytoskeleton rearrangement and cell adhesion in keratinocytes (152, 157, 158) For instance, the activation of p38 mitogen-activated protein kinase (p38MAPK) plays a vital role in causing the loss of cell cohesion. Blocking p38MAPK in the human epidermis has been shown to prevent blistering. Therefore, the specific morphological alterations induced by pathogenic IgG in mucocutaneous PV such as widening between desmosomes and the decrease in desmosome size are at least partially associated with p38MAPK signaling (152, 157). Monovalency resulting from Fab-arm exchange was shown to increase the pathogenic effect of patient-derived autoantibodies in PF (159). The effect of valency in PV seems less pronounced (160, 161). Monovalent single-chain variable-region fragments of autoantibodies derived from PV patients’ demonstrated pathogenic capacity *in vivo* (151) as well as Fabs of patient-derived mAbs (161). In pemphigus, there is no direct correlation of autoantibody titer to disease severity and as such, the titer cannot be used to monitor disease activity directly (162, 163).

### 4.4 LGI1/CASPR2-antibody Encephalitis

Antibody-mediated encephalitis is a heterogeneous group of disorders caused by more than 21 different antibodies (164). Among these, anti-LGI1 and anti-CASPR2 encephalitis are related to IgG4 as the dominant immunoglobulin subtype (165). LGI1 is a synaptic protein that binds to presynaptic metalloproteinase domain-containing protein 23 (ADAM23) and postsynaptic ADAM22. ADAM23 positions voltage-gated potassium channels in the presynaptic terminal and AMPA receptor in the postsynaptic membrane. Antibody binding disrupts LGI1-ADAM22/ADAM23 complexes on the cell surface leading to diminished AMPAR and voltage-gated potassium channel (VGKC) clusters. Loss of inhibitory VGKC complexes heightens neuron excitability, while AMPAR loss impairs long-term potentiation. The AMPAR loss is believed to directly contribute to the memory deficits observed in individuals with anti-LGI1 encephalitis (166, 167). CASPR2 serves as a transmembrane cell adhesion protein that interacts with Kv1.1 and Kv1.2 VGKCs in the juxtaparanodal region of myelinated peripheral nerves. In addition to its distribution in the nodes of Ranvier across the central and peripheral nervous systems, CASPR2 is also found in the synapses of the limbic system and basal ganglia (164). In mice, intrathecal infusion of anti-CNTN2 IgG, comprising a mixture of IgG1 and IgG4, purified from individuals with anti-CASPR2 encephalitis, were observed to induce memory deficits. This effect was attributed to hindering CASPR2/TAG1 interaction and reducing the surface levels of CASPR2, Kv1.1, and AMPAR, similar to LGI1 antibodies (168). CASPR2 antibodies exert their pathogenicity mainly through blocking (169), while studies showing the effect of monovalency on pathogenicity are currently missing. In LGI1 encephalitis the effect of valency on the pathogenic capacity has also not been investigated in detail yet. Similarly, the correlation of the autoantibody titer in the bloodstream with clinical disease severity is unknown. Levels of autoantibodies found in the cerebrospinal fluid might serve as a more accurate indicator in these pathologies of the central nervous system.

### 4.5 IgG4-RD

IgG4-RD is an immune-mediated systemic condition characterized by fibroinflammatory lesions in various organs; these lesions can mimic malignancies, infections, and inflammatory disorders, often accompanied by elevated IgG4 levels, though not always (170, 171). Recognized as a distinct disease only since 2003, early diagnoses of IgG4-related disease were often incidental findings during surgical resections of lesions initially suspected to be malignant (172, 173). IgG4-RD is characterized by three major histopathological findings: a dense lymphoplasmacytic infiltrate, fibrosis, at least focally in a storiform pattern, and obliterative phlebitis. Additionally, a diagnosis requires an increased number of IgG4 plasma cells in the tissue (174). The 2019 American College of Rheumatology/European League Against Rheumatism (ACR/EULAR) established comprehensive criteria for diagnosing and investigating IgG4-RD. These criteria include: (1) involvement of at least one of 11 possible organs, (2) a total of 32 exclusion criteria, and (3) eight weighted inclusion criteria (175).

Regarding the underlying immunopathology, CD4+ cytotoxic T lymphocytes play a pivotal role, constituting a major subset in both tissue and circulation. These cells secrete pro-fibrotic cytokines such as IL-1β, TGF-β1, and IFN-γ, along with cytolytic molecules like granzymes (176, 177). Among this population, the dominant effector subset is characterized by CD27lo CD28lo CD57hi cells with clonal expansion, and activated CD8+ T cells expressing granzyme-A are also observed (178). A recent study on tertiary lymphoid organs in IgG4-RD revealed a significant infiltration of a Tfh subset that is LAG3 and IL-10 positive (61). Activated B cells and plasmablasts may interact with these CD4+ T cell subsets, contributing to fibrosis and inflammation in IgG4-RD (176). Notably, a study highlighting dominant plasmablast clones identified galectin-3 autoantibodies, present in a subset of patients and correlated with galectin-3 plasma levels (179). Unlike autoantibody-mediated autoimmune disorders, the direct involvement of B cells in IgG4-RD pathology remains unclear and requires further investigation. The circulating IgG4 antibodies in IgG4-RD most likely are predominantly monovalent due to Fab-arm exchange. Interestingly, the levels of IgG4 subclass antibodies is not always elevated in these patients (170, 171).

## 5 Therapeutic Interventions

The standard treatment for autoimmune diseases previously involved broad immunosuppressive drugs (180). While these therapies improve symptoms, their effectiveness is often limited by adverse side effects. Moreover, not all patients respond to these conventional treatments. Interestingly, patients who do not respond to standard therapies have shown positive responses to treatments originally used in B cell malignancies, such as anti-CD20 mediated BCDT (3–12). Studying the efficacy of anti-CD20 mediated BCDT has provided valuable insights into potential therapies for B cell pathologies. Additionally, distinct therapies influence the B cell repertoire differently as observed in patients that received mycophenolate mofetil and anti-CD20-mediated BCDT (181), highlighting the potential benefits of combination therapy. In this chapter, we will first examine anti-CD20 mediated BCDT and then explore new therapeutic approaches.

### 5.1 Anti-CD20-mediated BCDT

Originally developed for treating B cell malignancies, anti-CD20 mediated BCDT has proven effective in managing diverse autoimmune conditions, including multiple sclerosis, PV, rheumatoid arthritis, CIDP, and MuSK MG (124, 135, 182–184). Anti-CD20 mediated BCDT exhibits remarkable efficiency in IgG4-AID and IgG4-RD (3–12).

Many MuSK MG patients enter stable remission for several years following anti-CD20 mediated BCDT, with significantly reduced or non-detectable MuSK autoantibody titers (124, 125, 185). However, relapses can occur in some patients over time (68, 185–187). In these cases, the MuSK autoantibody titer may increase months before clinical-detectable relapse (114, 125, 127). During relapse, frequencies of plasmablasts and memory B cells are elevated, with disease-related autoantibody-expressing B cells identified within these populations (111, 113, 114, 188–190). Consequently, MuSK MG patients treated with anti-CD20 mediated BCDT undergo cycles of remission followed by phases of clinical relapse. Most patients with IgG4-mediated nodo-paranodopathies are unresponsive to IVIg and steroids, unlike classical CIDP. As such, IVIg and steroid unresponsiveness should lead to antibody testing in CIDP patients. Anti-CD20 mediated BCDT shows beneficial effects in the majority of patients with IgG4-mediated nodo-paranodopathies, particularly those refractory to other therapies (191–195). In a large cohort of anti-NF155 antibody positive patients, 77% of patients responded to anti-CD20 mediated BCDT (194). In addition, serum neurofilament light chain (NfL) levels which are an indicator for axonal damage and anti-NF155 antibody titers were also decreased after therapy. Similar positive outcomes have been observed in pemphigus (196–198). High-dose anti-CD20 mediated BCDT therapy extended remission duration, although it did not impact the relapse rate (40%) compared to a low-dose regimen (197). Furthermore, treatment response to anti-CD20-mediated BCDT is good in LGI1/CASPR2-antibody encephalitis (11, 199). Interestingly, anti-CD20-mediated BCDT has emerged as a highly effective therapy option in IgG4-RD, underscoring the potential involvement of B cells in the pathophysiology (13).

However, it is crucial to emphasize that the response to anti-CD20 mediated BCDT varies and not all patients achieve remission (125, 197, 200, 201). In MuSK MG, non-response in patients might be associated with a prevalence of pathogenic IgG1-3 subclass antibodies (110). Additionally, in AChR MG, which was previously considered a less favorable target for anti-CD20 mediated BCDT, a small subset of patients’ benefits from anti-CD20 mediated BCDT. These positive responses have been observed in cases of severe refractory AChR MG (200, 202). Anti-CD20 mediated BCDT appears to yield better results when initiated during the early stages of AChR MG (203, 204). This variability in response is likely due to the heterogeneity of the underlying immunopathologies (59) and an increasing significance of long-lived plasma cells in the immunopathogenesis as the disease progresses over time. In some instances, disease progression occurred post-anti-CD20 mediated BCDT administration (205), potentially linked to the presence of anti-RTX antibodies, known to impede responsiveness of anti-CD20 mediated BCDT in IgG4-mediated nodo-paranodopathies (206). Ocrelizumab, another BCDT targeting CD19 with lower immunogenic potential, has proven to be a successful alternative in patients developing anti-RTX mAbs (207). The variation in response may, to some extent, be associated with IgG subclasses other than IgG4 playing significant roles in the immunopathology of these patients (110).

**Figure 2:**
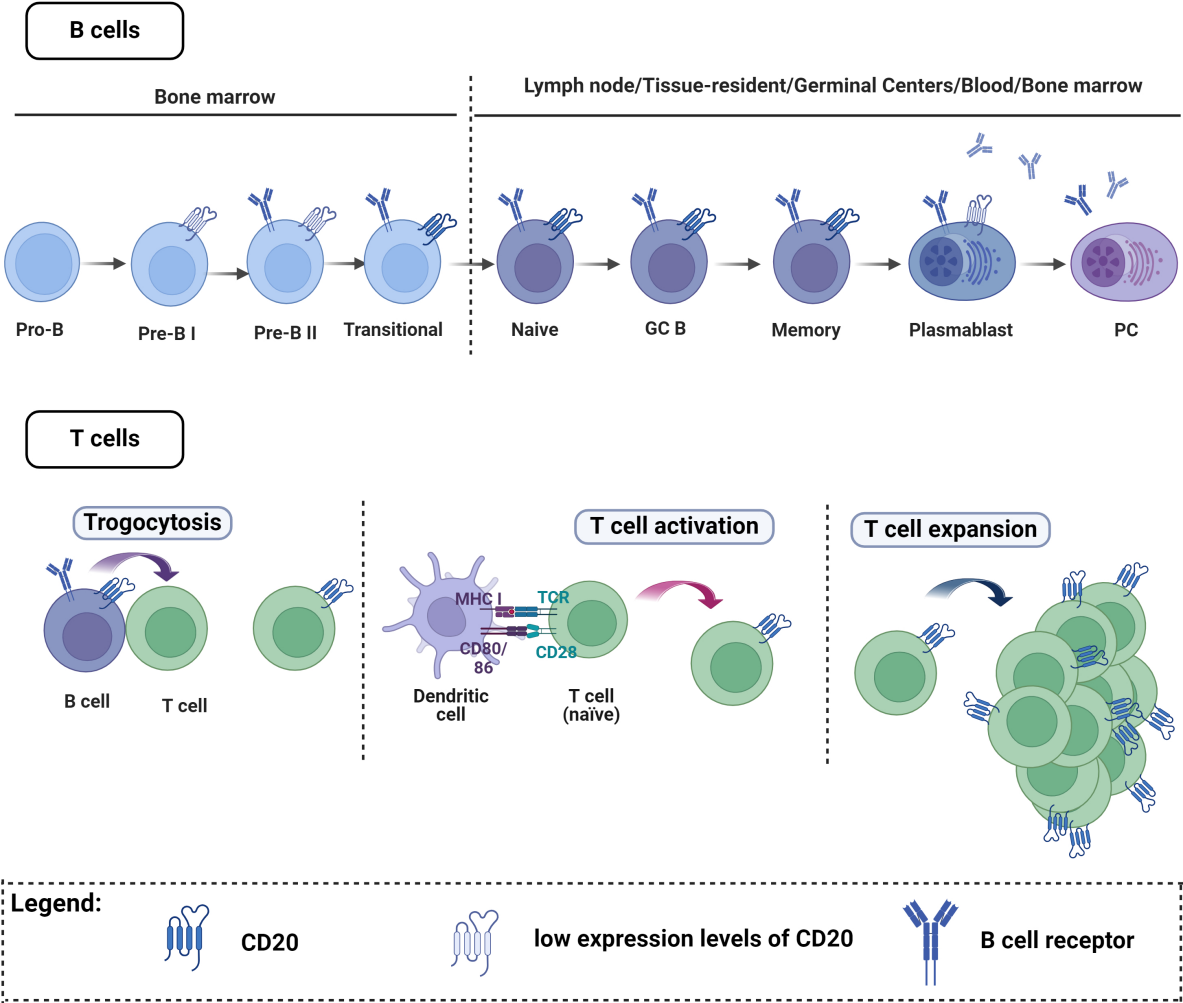
CD20 expression on B cells and T cells. B cells. CD20 is expressed at almost all stages of B cell maturation, with the exception of plasma cells, Pro-B cells, and Pre-B-I cells. T cells. A small number of T cells express CD20, and there are three main theories explaining how these cells acquire CD20: One explanation proposes that T cells may acquire CD20 through trogocytosis, involving simultaneous transfer from B cells. Alternatively, CD20 acquisition might occur during T cell activation or result from the clonal expansion of CD20-expressing T cells. Figure created with Biorender.com.

However, the complexity of CD20 expression should be acknowledged, extending beyond B cells to include T cells (**Figure 2**). Thus, anti-CD20 mediated BCDT does not exclusively affect B cells; subsets of T cells expressing CD20 on their surface are also impacted by this therapy (208–210). This depletion of CD20-expressing T cells has been observed in multiple sclerosis (208, 211). For B cells, CD20 is expressed at nearly all stages of B cell differentiation, excluding plasma cells, Pro-B-cells, and Pre-B-I (**Figure 2**) (16). The initial report of T cells with CD20dim expression (208, 212), was first widely considered a flow cytometry artifact (213). Subsequently, this population was identified as a distinct T cell subset with both immune-regulatory and proinflammatory activities (208, 214). The frequency of CD20+ T cells in the peripheral blood of healthy individuals is relatively low (1-4%). However, these cells are increased under inflammatory conditions and enriched in tissues including tonsils, thymus, bone marrow, and cerebrospinal fluid compared to the peripheral blood (208, 210, 215, 216). The origin of CD20-expressing T cells remains unclear (**Figure 2**). One proposed explanation is that T cells acquire CD20 through the process of trogocytosis, involving the simultaneous transfer of HLA-DR and CD20 from B cells (210, 216–218). However, the low expression of HLA-DR on T cells suggests that trogocytosis may not fully elucidate the origin of this subset (208, 217). Additionally, CD8+ T cells are known to interact mainly with cross-presenting dendritic cells during conditions such as viral infection, rather than B cells. As dendritic cells are not known to express CD20 on cell surface, the acquisition of CD20 by CD8+ T cells through trogocytosis remains questionable. Moreover, *MS4A1*, the gene that encodes CD20, was shown to be transcribed in T cells (208). Therefore, CD20 acquisition may occur during T cell activation (217, 219) or result from clonal expansion of CD20-expressing T cells (217, 220). Myelin-specific CD8+ CD20+ T cells were shown to be depleted together with B cells after CD20 monoclonal therapies (221). The major T cell subtypes that were lost included memory CD8+CD20+ and central memory CD8+ T cells but not CD4+CD20+ T cells and this may contribute to increased infection rate seen in these patients (222). Another study found that pretreatment levels of CD8+CD20+ T cells that have a proinflammatory phenotype have a significant inverse relationship with disease burden before treatment. Additionally, these cells were predictive of early disease activity following the initiation of anti-CD20 therapy (223). Therefore, RTX’s effectiveness in these autoimmune diseases may not only be the consequence of RTX’s effect on B cells.

B cell depletion by anti-CD20 mediated BCDT is further limited, as disease-relevant B cells persist even after treatment (114, 190). The persistence of these B cell clones is not unique to MuSK MG; this phenomenon extends to various autoimmune disorders, including Sjögren’s syndrome, SLE, systemic sclerosis, and ANCA-associated vasculitis (181, 224, 225). These persistent B cells were found to reemerge in MuSK MG months before clinical detectable relapse simultaneously with an increase of autoantibody titer (114). Several characteristics distinguish persistent B cell clones. CD20 expression is crucial for the efficacy of anti-CD20-mediated B cell depletion therapy. Both persistent memory B cells and antibody-secreting cells (ASCs) express low CD20 levels (114, 190). Furthermore, persistent memory B cell subsets express genes associated with previous tissue homing (114, 188, 190, 226–228). Additionally, B cell memory clones that are highly expanded show a higher rates of persistence (190, 229–231). Alterations in the BAFF/APRIL system, including reduced BAFF-R expression and elevated TACI and BCMA levels, are observed in persistent clones and plasmablasts at the time of relapse (114, 190, 232). Nevertheless, it is evident that the remarkable effect of anti-CD20 mediated BCDT in IgG4-associated disorders suggests a direct impact on the reservoir of pathogenic cells in these diseases.

### 5.2 New therapeutic approaches

#### 5.2.1 Anti-CD19 mediated BCDT

CD19 is a transmembrane glycoprotein from the immunoglobulin superfamily (233) and it is functionally associated with modulation of antigen-independent B cell differentiation and immunoglobulin-induced B cell activation (234). The expression profile of CD19 is broader than that of CD20, starting earlier at the stage of pro-B cells and extending into plasmablasts and plasma cells beyond the expression of CD20 (234, 235). Two anti-CD19 mAbs have reached clinical trials: XmAb5871 and Inebilizumab (236, 237). XmAb5871 is currently investigated in a phase II clinical trial (ClinicalTrials.gov Identifier: NCT02725476) and Inebilizumab (ClinicalTrials.gov Identifier: NCT04540497) in ongoing phase III clinical trials for patients with IgG4-RD. Inebilizumab is further tested in MG (MINT; ClinicalTrials.gov Identifier: NCT04524273). CD19 has gained attention in recent years as an alternative therapy to anti-CD20 mediated BCDT. Given its broader expression profile, especially in ASCs. Besides, it could be an interesting alternative for patients developing resistance to CD20 therapies, as it has already been reported in some non-responders (207).

#### 5.2.2 Anti-CD38 mediated BCDT

The main expression of CD38 is observed in hematopoietic cells. It was first identified as a lymphocyte specific antigen, but current studies revealed that it is ubiquitously expressed (238, 239). Plasma cells and memory B cells express high levels of CD38, while it is expressed at low levels in normal lymph and bone marrow cells (240). CD38 is also expressed in B cell precursors, germinal center B cells, and plasma cells and in other immune cells like NK cells, neutrophils and myeloid cells (239). CD38 is a multifunctional, membrane-bound protein that serves as an antigen and as an enzyme. It catalyzes the metabolism of cyclic ADP-ribose (cADPR) and nicotinic acid adenine dinucleotide phosphate (NAADP). These are two different calcium second messengers, involved in several cell functions (238, 239). Besides this enzymatic function, CD38 can also act as a receptor for CD31, acting as an adhesion molecule for mediating selectin-like binding of hematopoietic cells to endothelial cells and facilitating their transmigration to tissue (239). The role of CD38 in immune cells ranges from immunomodulation to effector functions during inflammation, where it could regulate cell recruitment, cytokine release and NAD availability. This expression profile of CD38, together with its role in inflammatory processes, make CD38 an interesting target in the context of autoimmune diseases (239, 240). Antibodies targeting CD38 have been proposed as a potential therapeutic approach to eliminate plasma cells that produce autoantibodies. Daratumab and Isatuximab have already been established as an important therapeutic target for Multiple Myeloma (MM) (241). Isatuximab binds to an epitope that partially covers the catalytic site of CD38, without changing its configuration, while Daratumumab is binding outside the catalytic site (242). In SLE patients, Daratumumab was found to restore NK cytotoxic function which promoted the elimination of circulating plasma cells (240). Thus, anti-CD38 might be a potent drug to reduce the levels of ASCs.

#### 5.2.3 Anti-CD40 mediated BCDT

CD40 is constitutively expressed on B lymphocytes, and CD40L is mainly expressed in the surface of activated CD4+ T cells, inducing activation, proliferation and cytokine production. Besides, they are found in other hematopoietic cells such as monocytes and dendritic cells as well as non-hematopoietic cells like mast cells, basophils, NK cells, macrophages, megakaryocytes and platelets (243). CD40 is a receptor of the TNF superfamily (243) and together with its ligand, CD40L (CD154), they form an important stimulatory immune checkpoint (244). CD40/CD40L interaction is essential for the formation of germinal centers and the production of class-switched antibodies. It is also important in humoral and cellular immunity, being involved in the activation of innate and adaptative immune cells, regulation of B cell, T cell and APC activation and immunological memory (243, 244).

This role in activation of immune responses makes it an attractive target for potential therapies, and recently, it has been the subject of multiple studies with this aim (244). Iscalimab is a human anti-CD40 monoclonal antibody (244) and it has been recently evaluated for safety and efficacy in moderate to severe symptomatic MG in a phase II clinical trial (245). Previously, a therapeutic benefit had been reported in patients with other autoimmune diseases such as Sjörgen Syndrome (246) and Graves´disease (247). In the case of Pemphigus, the interaction between CD40 and its ligand is important for the induction of pathogenic anti-Dsg3 IgG antibodies (248). Therefore, anti CD40L could be a potential therapeutic approach for Pemphigus (249). In summary, antibodies directed against the CD40/CD40L axis constitute a novel target with potential benefits for patients with autoimmune diseases, by targeting plasma cells involved in the production of pathogenic autoantibodies.

#### 5.2.4 Targeting the BAFF/APRIL-system

The BAFF/APRIL-system plays a crucial role in regulating the survival and maintenance of B cells. This system consists of two key ligands, B-cell activating factor (BAFF) and a proliferation-inducing ligand (APRIL), along with three receptors: the transmembrane activator and calcium modulator and cyclophilin ligand interactor (TACI), B cell maturation antigen (BCMA), and B-cell activating factor receptor (BAFF-R) (250). BAFF and APRIL ligands are primarily produced by myeloid and stromal cells, while their corresponding receptors are found on circulating B cells. These receptors are expressed at various stages of B cell development, suggesting a dynamic regulation of B cell survival and maintenance throughout maturation (16). Dysregulation within the BAFF/APRIL-system is associated with autoimmune diseases as well as cancer, allergies, transplants, infections, and immunodeficiencies (251). Elevated levels of BAFF/APRIL soluble receptors and ligands are linked to B cell pathologies, observed in conditions like MG, SLE, Sjögren´s syndrome, and RA, among others (251–255).

This pivotal role in autoimmune diseases suggests that targeting components of this system may offer a potential therapeutic avenue (251). Belimumab (GSK1550188), a recombinant monoclonal antibody targeting soluble BAFF, was the first FDA-approved biologic for SLE treatment, following positive safety and efficacy outcomes in phase III clinical trials (ClinicalTrials.gov Identifier: NCT00424476; ClinicalTrials.gov Identifier: NCT00410384) (251). Unexpectedly, Belimumab did not demonstrate significant efficacy in a phase II clinical trial for Myasthenia Gravis (AChR and MuSK) (ClinicalTrials.gov Identifier: NCT01480596) (256). Other BAFF-targeting agents such as Blisibimod and Tabalumab have undergone phase II/III clinical trials for diseases like SLE and MM (257–259). In recent years, research has focused on dual inhibitor agents, like Atacicept, which inhibit both BAFF and APRIL simultaneously. Atacicept is designed as the extracellular domain of the receptor TACI fused to the human Fc domain (260, 261). Atacicept has shown beneficial effects in SLE and RA patients by significantly depleting plasma cells (262–266) and Telitacicept, another dual inhibitor, has also demonstrated positive effects in autoimmune diseases (259). In a phase II clinical trial for SLE, Telitacicept exhibited beneficial effects (267). Additionally, there is an ongoing phase III clinical trial in China to evaluate the efficacy and safety of Telitacicept in the treatment of MG, currently recruiting (ClinicalTrials.gov Identifier: NCT04660565). Another ongoing phase IV clinical trial in China is assessing the use of Belimumab for the treatment of IgG4-RD, with results pending (ClinicalTrials.gov Identifier: NCT04660565). Although dual inhibtion of BAFF and APRIL seems to have beneficial effects in some autoimmune disorder, in multiple sclerosis a trial for atacicept had to be halted due to increased disease activity in patients compared to placebo (268–270). Thus, the complexity of the BAFF/APRIL-system has to be further investigated to better understand how therapy influences the underlying immunopathology of individual diseases.

#### 5.2.5 Proteasome inhibitors

The ubiquitin/proteasome system constitutes one of the main pathways for intracellular protein degradation (271, 272). Therefore, it is a key component for maintaining a dynamic control over key signaling components of the immune response, as well as overall cell homeostasis. Malfunctioning of the proteasome system is associated with pathological conditions, like cancer or autoimmune disorders (272). Proteasome inhibition results in the accumulation of defective immunoglobulin chains, leading to stress in the endoplasmic reticulum, misfolding of proteins and ultimately cell apoptosis (271, 273). It has been proven to be critical for plasma cell function due to their high rate of antibody synthesis. Inhibiting the proteasome results in the apoptosis of plasma cells and a consequent decrease in antibody production. Besides, proteasome inhibition also hinders the production of pro-inflammatory cytokines (273). This makes the proteasome a promising novel target for antibody mediated autoimmune diseases, involving long-lived plasma cells, like SLE (273) or AChR-MG (59).

Bortezomib, initially approved for MM, is a dipeptide boronic acid derivative, that binds to the catalytic site of the proteasome with high affinity on plasma cells (272, 273). There have been several clinical trials for Bortezomib in SLE patients, that probe a remarkable efficacy of Bortezomib, especially in SLE patients that had been refractory to prior immunosuppression therapies (271, 274). Bortezomib and other proteasome inhibitors have shown beneficial effect in EAMG, a mouse model of MG (275, 276). *In-vitro* studies of thymic cell cultures derived from AChR-MG patients with Bortezomib could eliminate thymus-derived plasma cells and reduce IgG levels (277). A phase 2 clinical trial (ClinicalTrials.gov Identifier: NCT02102594) to investigate the use of Bortezomib on patients with therapy-refractory Myasthenia Gravis (generalized) or SLE or RA is currently still ongoing (278). Thus, Bortezomib is another good potential candidate for a more targeted immunotherapy in B cell pathologies.

#### 5.2.6 FcRn inhibitors

Human IgG is one of the most abundant proteins in serum, probably because of the uniquely long half-life, for which neonatal fragment crystallizable (Fc) receptor (FcRn) plays an essential role (279, 280). The FcRn is a multifunctional atypical form of Fc-gamma receptor. It was first identified as the responsible of transporting IgG from the maternal to the fetal circulation. Later it was found that, among other functions, it plays an essential role in IgG recycling, by protecting IgG from intracellular degradation, therefore, expanding the half-life (280, 281). FcRn inhibitors enhance the catabolism of IgG by blocking the FcRn-mediated IgG recycling pathway leading to reduced IgG levels in serum and a decrease in pathogenic autoantibodies (282).

In recent years, FcRn inhibitors have emerged as promising targets for autoantibody-mediated autoimmune diseases. Efgartigimod (ARGX-113) binds to FcRn, preventing IgG recycling (282). A phase III trial in MG showed efficacy and tolerability (283) and an ongoing trial for pediatric MG patients is currently recruiting (ClinicalTrials.gov Identifier: NCT05374590), alongside a phase III trial assessing different dosing regimens (ClinicalTrials.gov Identifier: NCT04980495). SYNT001, another FcRn inhibitor, demonstrated efficacy in a phase I/II trial for Pemphigus (284). Rozanolixizumab, also an anti-FcRn monoclonal antibody, showed positive results in MG clinical trials (285). Several trials are ongoing for Rozanolixizumab in MG treatment, including a phase III trial (ClinicalTrials.gov Identifier: NCT04650854) and a self-administration study (ClinicalTrials.gov Identifier: NCT05681715). Additionally, a phase II/III trial for pediatric MG patients is ongoing (ClinicalTrials.gov Identifier: NCT06149559). Other FcRn inhibitors like Batoclimab are also in clinical trials, including a phase III trial for generalized MG (ClinicalTrials.gov Identifier: NCT05403541) and testing in patients with active CIDP (ClinicalTrials.gov Identifier: NCT05581199). Overall, FcRn inhibitors hold promise as therapy for IgG-driven autoimmune diseases like MG, offering targeted treatment with potentially fewer side effects than nonspecific therapies.

#### 5.2.7 Complement inhibitors

The complement system comprises a network of over 30 proteins, which interact in a sequential and regulated manner, culminating in the formation of the membrane attack complex (MAC). The MAC inserts into cell membranes, leading to pore formation and cell damage (286). The classical complement pathway is activated by antibody-antigen complexes (see chapter 3). Not all antibody isotypes are capable of activating the complement cascade. Targeting the complement system in diseases with autoantibodies that are mainly of the IgG1 or IgG3 subclass has shown remarkable efficacy, especially in the context of AChR MG. AChR autoantibodies are mainly of the subclass IgG1 (287). Eculizumab, inhibiting complement activation by targeting C5, demonstrated beneficial outcomes in phase III trials (ClinicalTrials.gov Identifier: NCT02301624; ClinicalTrials.gov Identifier: NCT01997229) and is approved for treating AChR+ MG patients (288). Ravulizumab, another C5 inhibitor, is currently in phase III trials (ClinicalTrials.gov Identifier: NCT03920293) (289). However, complement inhibition may not provide clinical benefits for patients suffering from IgG4 mediated diseases.

#### 5.2.8 BTK inhibitors

Bruton´s tyrosine kinase (BTK) is a multifunctional cytoplasmic protein member of the TEC kinase family (290), crucial in B-cell biology, involved in maintaining B-cell survival, proliferation, differentiation and activation (291). Apart from B cells, it is also expressed in mast cells, NK cells, T cells, macrophages, neutrophils, monocytes and basophils (291), highlighting its multifaceted role in the immune system. Among the functions of BTK, it integrates signaling to regulate B cell development through BCR (290). Besides it is also involved in TLR-mediated signaling, chemokine mediated homing of pre-B cells into lymphoid organs and mediates in inflammatory processes driven by IgG complexes (291). Due to its central role in B cell immunity, BTK inhibition therapies have been gaining attention as first-line therapy for B cell malignancies (292). Its involvement in B cell survival and differentiation has also linked BTK with autoimmune diseases (291). Indeed, studies have shown increased levels of BTK expression in B cells from patients suffering from autoimmune diseases, which appears to be correlated with autoantibody production (293). Therefore, BTK inhibitors represent a promising therapeutic strategy for autoimmune diseases. BTK inhibitors are small molecules that are able to downregulate various B cell functions like cell proliferation, differentiation, maturation and survival overall. Besides, they can inhibit the activity of macrophages, mast cells and eosinophils (249). Currently, inhibition of BTK is being investigated for SLE (271). Fenebrutinib, a BTK inhibitor, was investigated in a phase II clinical trial in SLE (ClinicalTrials.gov Identifier: NCT02908100). Despite effectively targeting the BTK pathway, it did not meet the trial’s primary endpoint for efficacy (294). In pemphigus, the BTK inhibitor PRN1008 has been studied in a phase III clinical trial (ClinicalTrials.gov Identifier: NCT03762265), but it was terminated based on lack of efficacy. Although, the efficacy of BTK inhibitors as standalone treatments is limited, it might still hold potential in combination therapies.

#### 5.2.9 CAR and CAAR T cells

Chimeric antigen (CAR)-T cell therapies, initially designed for targeting tumor cells, involve priming T cells with anti-tumor activity and then reintroducing them into the patient (295–297). These modified T cells express chimeric antigen receptors (CARs), genetically engineered to have tumor antigen-specific binding sites and a T cell activating domain (298). This allows them to recognize and kill cells expressing the target antigen.

Anti-CD19 CAR T cell therapy gained FDA approval in 2017 for treating B cell malignancies (299), sparking interest in expanding CAR T cell applications beyond cancer, particularly in autoimmune therapies (300). CAR-T therapies aim to redirect T cells against autoantibody-secreting B cells. In addition to CAR T cells, chimeric autoantibody (CAAR) receptor T cell therapies were developed (301–305). CAAR T-cells, distinct from CAR T-cells, express chimeric autoantibody receptors targeting pathogenic antibodies from autoreactive B cells (306). These include anti-BCMA (301–304), investigated in the context of MG (ClinicalTrials.gov identifier: NCT04146051), anti-CD19 (303) (ClinicalTrials.gov identifier: NCT03030976), and most recently, anti-MuSK and anti-NMDA, which have shown initial beneficial effects in a mouse models (305, 307). Another phase I trial (ClinicalTrials.gov Identifier: NCT04422912) is assessing Dsg3-CAAR T cells in Pemphigus vulgaris (308). CAR and CAAR T-cell therapies constitute a promising approach for a more selective and personalized treatment of autoimmune diseases.

## 6 Conclusion and outlook

The reason why some autoimmune diseases are predominantly IgG4 and not another subclass is still not completely understood. Despite the shared characteristic of having autoantibodies belonging to the IgG4 subclass, these diseases exhibit numerous differences. No discernible pattern emerges concerning the site of pathology, age of onset, or specific genetic predispositions (**Table 1**). Generally, the pathogenesis of autoimmune disorders is the result of a combination of defects in the immune system together with environmental, and genetic factors similar to the observed pathogenesis of multiple sclerosis, SLE, and type 1 diabetes (309–313). Given the rarity of some of these diseases, our current understanding may be limited. Thus, it is highly probable that clear patterns are not yet discernible. Future investigations are likely to provide more clarity.

IgG4 antibodies play dual roles in health and disease (49). In health, they serve protective and regulatory functions by predominantly inhibiting the actions of IgE antibodies (45, 47–49). However, in autoimmune conditions, where IgE antibodies are not the primary effectors, IgG4 antibodies contribute directly to immunopathology. Similarly, Fab-arm exchange seems to be a coincidental aspect of IgG4-mediated diseases. Fab-arm exchange enhances the diversity of IgG4 antibodies and increases their potential to engage with antigens (45, 47–49). This intrinsic property of IgG4 antibodies can either enhance the pathogenicity of autoantibodies, as observed with MuSK autoantibodies (112, 121–123), without significantly affecting their effects as seen in CNTN1 autoantibodies and pemphigus (142, 151, 161) or diminish their pathogenic potential as seen with anti-Neurofascin-155 IgG4 (141). Therefore, there is a variability in the relevance of Fab-arm exchange for pathogenic capacity in these diseases. Nevertheless, the underlying immunopathology in these IgG4-mediated diseases appears to share similarities, as seen by the overall positive response to anti-CD20-mediated BCDT in these diseases (3–12). Hence, it is probable that comparable treatments will yield similar outcomes in these conditions.

## Author contributions

SÜ: Writing – review & editing, Writing – original draft, Conceptualization. BS: Writing – review & editing. EÇ: Writing – review & editing, Writing – original draft, Conceptualization. DB: Writing – review & editing. RMH: Writing – review & editing. SV: Writing – review & editing, Writing – original draft, Conceptualization. AV: Writing – review & editing, Writing – original draft, Conceptualization. AM: Writing – review & editing, Writing – original draft, Conceptualization. MF: Writing – review & editing, Writing – original draft, Visualization, Supervision, Conceptualization.
